# Mental Health Assessment of the Population: Study Protocol of the MAP Research Program in Ukraine (MAP-U) and in Zurich (MAP-Z)

**DOI:** 10.3389/ijph.2024.1607271

**Published:** 2025-01-13

**Authors:** Viktoriia Yasenok, Eileen Neumann, Alessia Raineri, Julia Kopp, Seraina Rüegger, Tala Ballouz, Marco Kaufmann, Andrii Loboda, Vladyslav Smiianov, Andreas M. Baumer, Erich Seifritz, Heiko Fabian Königstein, Anja Frei, Viktor Von Wyl, Susi Kriemler, Andriana Kostenko, Milo A. Puhan

**Affiliations:** ^1^ Epidemiology, Biostatistics and Prevention Institute, University of Zurich, Zurich, Switzerland; ^2^ Department of Adult Psychiatry and Psychotherapy, University Hospital of Psychiatry Zurich, Zurich, Switzerland; ^3^ Sumy State University, Sumy, Ukraine; ^4^ Institute for Implementation Science in Healthcare, University of Zurich, Zurich, Switzerland; ^5^ GFA Consulting Group GmbH, Hamburg, Germany

**Keywords:** mental health, war-affected population, post-traumatic stress disorder, depression and anxiety, alcohol use disorder

## Abstract

**Objectives:**

To conduct mental health surveillance in adults in Ukraine and Ukrainian refugees (Canton of Zurich, Switzerland) as an actionable scientific foundation for public mental health and mental healthcare.

**Methods:**

Mental Health Assessment of the Population (MAP) is a research program including prospective, population-based, digital cohort studies focused on mental health monitoring. The study aims to include 17,400 people from the general population of Ukraine, 1,220 Ukrainians with refugee status S residing in the canton of Zurich, and 1,740 people from the Zurich general population. The primary endpoints are prevalence and incidence of symptoms of: posttraumatic stress disorder (measured by PCL-5), depression (PHQ-9), anxiety (GAD-7), and alcohol use disorder (AUDIT). Secondary endpoints include participants’ health-related quality of life (EQ-5D-5L and EQ-VAS), experiences of somatic distress syndrome (PHQ-15), social isolation, social integration, and mental wellbeing (SWEMWBS).

**Results:**

Baseline assessment starts in March 2024 with follow-ups occurring every 3 months for at least 2 years.

**Conclusion:**

MAP will generate reliable, comparable, and high-quality epidemiological data to inform public mental health and healthcare policies in the Ukrainian population.

**ISRCTN Registry:**

https://www.isrctn.com/ISRCTN17240415.

## Introduction

Mental health challenges are pervasive and long-lasting in post-war societies, often associated with intra-familial violence or substance abuse [1]. Even before the Russian invasion in 2022, Ukraine already had a high mental health burden. Some of the drivers for this burden include historical Soviet-era trauma, Russia’s invasion of Ukraine in 2014, and, more recently, COVID-19 [2, 3].

Prior to the war, the prevalence of most mental health conditions in Ukraine was similar to those in Eastern Europe. However, the prevalence of alcohol use disorders was notably higher in Ukraine (6.0%) compared to the global average (1.5%) [4]. The current, unmeasured, public mental health crisis in Ukraine likely began before the start of the war with the Russian occupation in 2014. A study from 2016 on mental health of internally displaced persons in Ukraine (due to the armed conflict in the east of Ukraine) estimated a prevalence of 32% for posttraumatic stress disorder (PTSD), 22% for depression, and 17% for anxiety [5], which is several times higher than the Eastern European average. A further analysis found that 55% of respondents were at risk for developing a somatic symptom disorder [6] and 8.4% of men as well as 0.7% of women tested positive for alcohol use disorder [7].

According to WHO global estimates, one in five people living in an area affected by conflict at any time during the previous 10-year period is estimated to have some form of mental health condition, ranging from mild depression or anxiety to even psychosis. Almost one in 10 is expected to live with a moderate or severe mental health condition [8]. Applying these estimates to the population of Ukraine (about 43.7 million) [9], approximately 9.6 million people may live with a mild mental health condition and 3.9 million with a moderate or severe mental health condition. In addition to these projections that reach the diagnostic criteria of mental health conditions, an even larger proportion of the population is likely to experience distress, with common presentations such as feelings of anxiety and sadness, hopelessness, difficulty sleeping, fatigue, irritability, or anger [10].

While the long-term, population-level mental health impact of Russia’s invasion of Ukraine since 24 February 2022, cannot yet be fully predicted, increased demand for mental health and psychosocial support is anticipated both during the ongoing war and in a post-war context. Mental wellbeing of the Ukrainian population is key for societal and economic recovery and for long-term development. Some early work has assessed some mental health outcomes cross-sectionally [11–13] but no ongoing longitudinal mental health surveillance program in Ukraine is in place. Consequently, neither the public nor health authorities are informed in a timely manner about mental health needs of the population or the consequences of war activities on mental health conditions and health behaviours. Furthermore, mental health support, care needs, and acceptance are likely to vary across subgroups, for example, depending on the level of exposure to front-line violence (i.e., place of residence), and across vulnerable populations. Mental health surveillance programs to determine population and subgroup level mental health needs are essential to inform planning and allocation of services by the Ukrainian healthcare system, development partners, and both national and international organizations, including the International Committee of the Red Cross (ICRC). In the mid- and long-term, it is important to monitor the temporal development of mental health of the Ukrainian population and especially vulnerable subgroups to efficiently allocate resources for the large, expected need for community and mental healthcare.

Here, we describe the protocol of Mental Health Assessment of the Population (MAP), a research program consisting of longitudinal studies on mental health monitoring of the Ukrainian population. These studies are conducted across different regions in Ukraine and in the Canton of Zurich, Switzerland. The aim is to generate reliable data to inform public mental health and healthcare planning and evaluation.

### Study Objectives

The main goal of the MAP program is to implement an agile, digital surveillance of public mental health in adult people living in different parts of Ukraine and in Ukrainian refugees living in the Canton of Zurich, Switzerland. The specific objectives are: (1) to determine the prevalence of symptoms of PTSD, depression, anxiety, and alcohol use disorder (a) at the beginning of the surveillance, and (b) up to at least 2 years; (2) to determine short-term and long-term temporal developments of mental health in the Ukrainian general population (in Ukraine and in the Canton of Zurich) and subgroups defined by age and level of war-related exposure; (3) to assess how mental health impacts general health and healthcare seeking behaviour; and (4) to assess needs, preferences, barriers, and facilitators for mental healthcare services of the Ukrainian general population and subgroups.

## Methods

The protocol has been developed by an interdisciplinary and international team of Ukrainian and Swiss scientists, supported by stakeholders from universities and organizations in both countries. These include members of the ICRC, representatives from the University Hospital of Psychiatry Zurich, the Paediatrics Children’s Hospital of Zurich, and public health officials from Ukraine and Switzerland. It also involves experts in public and mental health, political science, health policy, and representatives from governmental (Ukrainian and Swiss) and non-governmental organizations (e.g., ICRC, UNESCO), as well as social media specialists.

### Study Design

The overarching MAP research program includes two related prospective, population-based, digital cohort studies: the Mental Health Assessment of the Population in Ukraine (MAP-U), which focuses on adult people living in Ukraine, and the Mental Health Assessment of the population in Zurich (MAP-Z), focusing on forcibly displaced Ukrainian citizens (refugee status S) living in the Canton of Zurich in Switzerland. MAP-Z also includes a control group of people from the general population of the Canton of Zurich not directly affected by the war. To maximize comparability between MAP-U and MAP-Z, we will use the same mental health surveillance protocol.

The project is conducted by the Epidemiology, Biostatistics and Prevention Institute (EBPI), University of Zurich (UZH), and Sumy State University, whereas the Embassy of Ukraine in Switzerland, Ministry of Health Ukraine, Center for Public Health Ukraine, as well as the Swiss partners Mental Health for Ukraine (MH4U) and the Swiss Agency for Development and Cooperation are cooperation partners.

The protocol has been approved by the responsible ethics committee of the canton of Zurich, Switzerland (Kantonale Ethik-Kommission Zürich; BASEC-Nr. 2023-02247) and by the Commission on Bioethics of Sumy State University, Ukraine (ref-Nr. 60-0274). The protocol has been prospectively registered on the International Standard Randomised Controlled Trial Number (ISRCTN17240415).

Upon enrolment, participants will complete the baseline assessment through a secure digital study platform (REDCap) [14, 15]. To access REDCap for the baseline and follow-up assessments, randomly selected persons in Ukraine will receive an email (automated, including reminders) containing a link to the respective assessment. If there is no response, we will approach them via Telegram or phone calls. For MAP-Z, the initial contact will be established by postal mail which has been proven to be a successful way in other population-based studies. Follow-up assessments, where participants will be invited by email or Telegram, will take place every 3 months for at least 2 years. The study design thus follows that of the digital cohort of Corona Immunitas, the COVID-19 Social Monitor and the Swiss MS Registry [16, 17]. Such a digital approach has the advantage of being independent from geography, thereby increasing the catchment area of interest [16, 17]. Participants from the Ukrainian population who are unable to fill out the questionnaires online are offered to complete the questionnaire through a phone interview (MAP-U only). As with the COVID-19 social monitor, results from MAP will be displayed in almost real-time on an online platform, providing the public, policymakers and scientists with the opportunity to monitor the prevalence and trajectories of symptoms of PTSD, depression, anxiety, and alcohol use disorder among the general population and by relevant subgroups.

### Primary Endpoints

The primary endpoints are the prevalence and incidence of symptoms of PTSD, depression, anxiety, and alcohol use disorder. Since we will not be able to establish diagnoses through mental healthcare experts (psychiatrists or psychologists) according to the International Classification of Disease (ICD)- 10/11, or the Diagnostic and Statistical Manual of Mental Disorders (DSM)- 5, we rely on commonly used, standardized questionnaires which we selected following a systematic selection process [18]. For the four mental health conditions of interest, we identified potential instruments and compared them against our selection criteria: (a) should have strong psychometric properties (construct validity, internal consistency reliability, test-retest reliability, and ability to detect change over time) to capture symptoms; (b) estimates of sensitivity and specificity are available (c) can be implemented in digital surveillance (i.e., can be completed independently by participants); (d) are available in the Ukrainian language; (e) are appropriate in the context of the war in Ukraine; (f) have established cut-offs for the presence or likely presence of the four conditions of interest; (g) are or have been used in other population-based studies; (h) are short in duration or low burden to complete; (i) have been previously used in Ukraine. Instruments were discussed and final decisions made at an international working group meeting held in Zurich in Spring 2023.

Based on the criteria mentioned above, we selected the Posttraumatic Stress Disorder Checklist (PCL-5) for the assessment of PTSD, Patient Health Questionnaire (PHQ-9) for depression, generalized anxiety disorder 7 (GAD-7) for anxiety, and Alcohol Use Disorders Identification Test (AUDIT) for the alcohol use disorder. References for each of the instruments regarding the validation of psychometric properties (in a general non-Ukrainian population), the translation process into Ukrainian, and validation and/or utilisation in the Ukrainian population are provided in Table 1. Furthermore, the selected instruments are aligned with those used by the Global Burden of Disease (GBD) from The Institute for Health Metrics and Evaluation.

**TABLE 1:** Mental Health Assessment of the Population, Switzerland, 2024

Instrument	Sensitivity %/Specificity % (score of instrument as cut-off)	Psychometric properties	Translation process	Validation/utilisation in Ukraine
PCL-5	94/94 (31)	Geier et al. [19] Karachevskiy et al. (2016) [20]	Roberts et al. (2019) [5]	Roberts et al. (2019) [5] ^a^ Karachevskiy et al. (2016) [20]
PHQ-9	88/88 (10)	Kroenke et al. (2001) [21]	Roberts et al. (2019) [5]	Roberts et al. (2019) [5] Osokina et al. (2022) [22] ^b^
GAD-7	89/82 (10)	Spitzer et al. (2006) [23]	Roberts et al. (2019) [5]	Roberts et al. (2019) [5] Osokina et al. (2022) [22] Ochnik et al. (2021) [24] ^b^
AUDIT	65/85 (5)	Moehring et al. (2019) [25]	Ramachandran et al. (2019) [7]	Ramachandran et al. (2019) [7] ^c^ Ministry of Health Ukraine (2014) [26] ^d^ Decyk et al. (2013) [27] Fitkalo (2023) [28]

*Note*:

^a^ Good reliability and construct validity in a Ukrainian sample.

^b^ Utilisation in a Ukrainian sample, no report on validation.

^c^ Good reliability in a Ukrainian sample.

^d^ Approval and recommendation of the Ukrainian version in a clinical context (alcoholic hepatitis) as a screening tool for measuring alcohol abuse.

Standardized instruments are often used for screening purposes with thresholds for the presence of a specific mental health condition that are optimized for sensitivity. As a consequence, prevalence is often overestimated when not corrected for accuracy [18]. Therefore, we will correct prevalence estimates for misclassification as described below.

We consulted systematic reviews [29–32] to assist us in identifying studies in the available literature that provide estimates of diagnostic accuracy in samples that are comparable to our study population. We selected estimates of sensitivity and specificity from studies if, whenever available, the studies included a population-based sample, used structured diagnostic interviews as reference standard, had a sample size of >100 and >50 positive cases, showed mean values that are comparable to those in our population (based on participants included up to June 30, 2024), and where we deemed the study population similar to our study population. We favoured primary studies over meta-analyses, because we are better able to assess similarity between our population and a single well-characterized sample than to compare our population with the heterogeneous aggregate of samples that underly a meta-analysis.

For the PHQ-9 we chose a cut-point of >=10 and assume a sensitivity of 0.88 and specificity of 0.88, as reported by Kroenke and colleagues [21]. The cut-point for the GAD-7 is >=10, the assumed sensitivity is 0.89 and specificity is 0.82 as reported by Spitzer et al. [23]. In the case of the AUDIT, we believe that the most applicable estimate of diagnostic validity was provided by Moehring et al. [25] based on a general population study in Germany. They observed a very similar AUDIT score distribution to the one in our population and, given a cut-point of >=5, reported a sensitivity of 0.65 and specificity of 0.85. The most applicable estimate for the diagnostic validity of the PCL-5 was provided by Geier et al. [19] which found that a cut-point of >=31 resulted in a sensitivity of 0.94 and a specificity of 0.94.

### Secondary Endpoints

Secondary endpoints include participants’ health-related quality of life (as measured by the European Quality of Life 5 Dimensions (EQ-5D-5L) instrument and the visual analogue scale [EQ-VAS]), experiences of somatic distress syndrome (measured with PHQ-15). To measure social isolation and social integration we adapted questions taken from the Swiss Health Survey (2022). Final English and Ukrainian versions of the Swiss Health Survey questions were developed through a process of duplicate independent translations followed by creating consensus-derived, culturally sensitive final versions. We use the Short Warwick-Edinburgh Mental Wellbeing Scale (SWEMWBS) to assess the mental wellbeing of the population. Needs, preferences, barriers, and facilitators for mental health services will be assessed by free-text questions.

Primary endpoints will be collected at each assessment timepoint. Secondary endpoints will be collected both at baseline and during follow-up. Follow-up assessment of secondary endpoints will be distributed over time (e.g., some at 3 months, some at 6 months), to shorten the length of follow-up assessments. Assessment of specific secondary endpoints at flexible time points depending on current events in Ukraine will also be considered. Prior to the final roll-out of the baseline and repeated assessments, we will conduct a small pilot study and focus group discussion with a diverse group of participants, to ensure the appropriateness of the assessments to the Ukrainian context and to the experiences of the population.

### Project Population, Inclusion, and Exclusion Criteria

#### MAP-U

**FIGURE 1:**
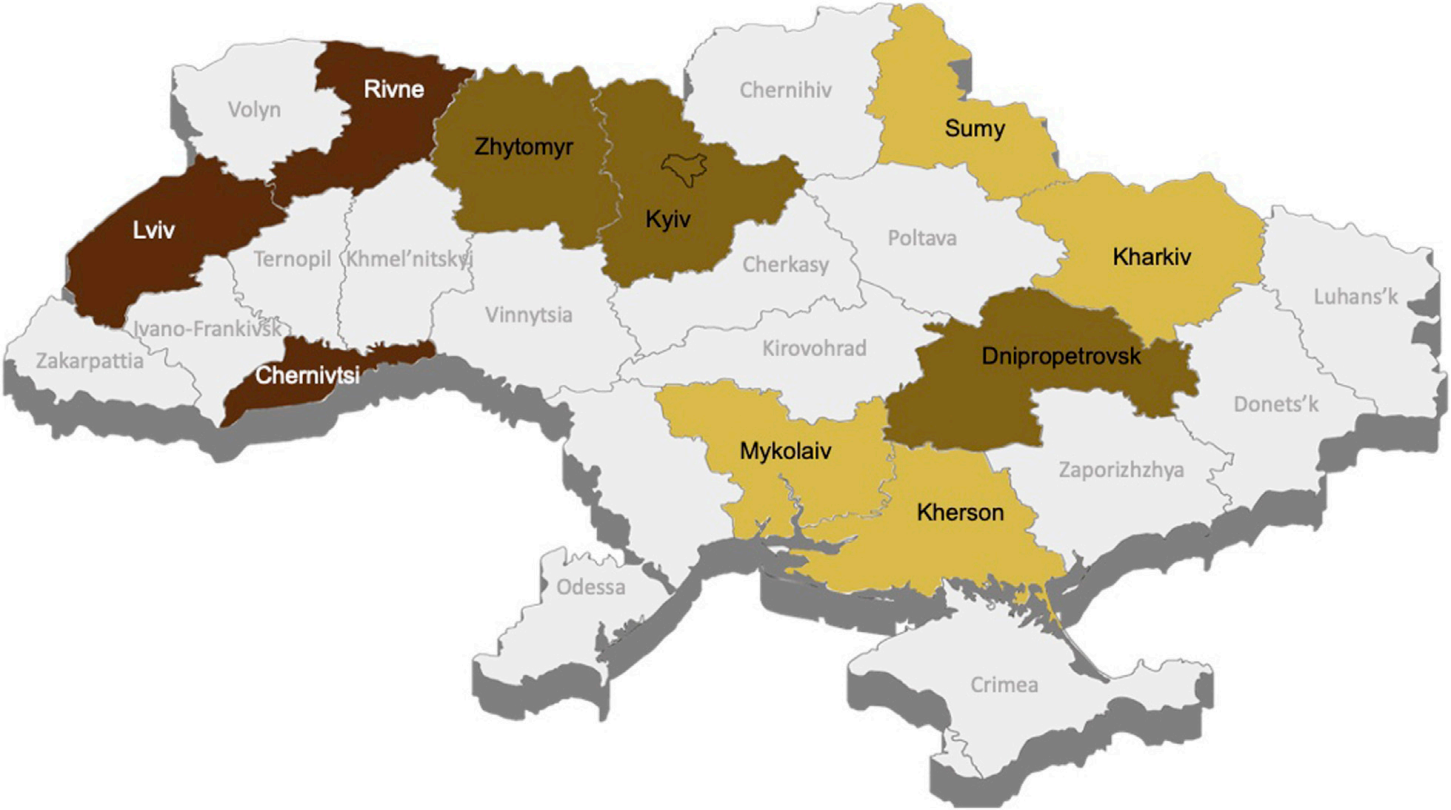
Mental Health Assessment of the Population, Ukraine 2024. Note. ^1^ in accordance with the Resolution of the Cabinet of Ministers of Ukraine dated October 14, 2022, No. 1159 “On approval of the Procedure for the development, holding of public discussion, approval of programs for the comprehensive restoration of the region, the territory of the territorial community (its parts), and making changes to them.” Access mode: https://zakon.rada.gov.ua/laws/show/1159-2022-%D0%BF#Text). Date of assessment: 15 June 2024.

The target population of MAP-U is adults (age 18 and over) living in Ukraine. The source population is adults registered as residents in 10 selected oblasts from all parts of the country that are affected differently by the war. Of the 27 oblasts of Ukraine, the 3 occupied oblasts (Figure 1; Luhansk, Crimea, Donetsk) are currently excluded from consideration. Specifically, the oblasts of Ukraine were randomly selected according to a geographical criterion of two oblasts from each region: Northern Ukraine (Zhytomyr, Rivne); Eastern Ukraine (Sumy, Kharkiv); Southern Ukraine (Mykolaiv, Kherson); Western Ukraine (Lviv, Chernivtsi); Central Ukraine (Kyiv, Dnipropetrovsk).

Because of the unstable situation in specific oblasts, we decided not to focus on individual oblasts for the analyses but on regional clusters of oblasts, defined by i) level of proximity to the hostilities zone/border with the aggressor = country-aggressor Russian Federation ii) consequences of the full-scale Russian invasion - level of destruction and loss of life; iii) geographical factors.

Cluster North-West (Rivne, Lviv, Chernivtsi oblasts): North-western Ukraine. These oblasts are remote from the hostilities zone and have low security risks due to their proximity to the EU border. There were no direct hostilities in these territories; therewas no large-scale destruction of infrastructure or housing due to hostilities; there has been significant population movement to this region.

Cluster Central (Zhytomyr, Kyiv, Dnipropetrovsk oblasts): Central Ukraine. These oblasts are far from the border with the aggressor and have medium security risks. Part of the territory experienced hostilities in the first months of the full-scale invasion; some areas were temporarily occupied or surrounded; there was destruction of infrastructure and housing due to hostilities and/or shelling.

Cluster South-East (Sumy, Kharkiv, Mykolaiv, Kherson oblasts): South-eastern Ukraine. These oblasts are close to the hostilities zone and have high security risks due to their proximity to the border with the aggressor. Most of the territory experienced hostilities, which are currently ongoing; a large part of the territory was temporarily occupied, and some settlements remain under occupation; significant destruction of infrastructure and housing stock due to hostilities and/or shelling is present; there has been mass emigration of the population to other regions and/or countries and a sharp deterioration in the level of socio-economic development.

Participants from these oblasts will be selected by an age-stratified (18–24, 25–44, 45–64, and 65+ years of age) random sample based on a database provided to the Center for Social Research by the State Statistics Service of Ukraine.

Inclusion criteria for the random sampling: adults (aged ≥18 years) who are residents of the participating oblasts as specified above, providing informed consent and are able to complete the surveys (available in Ukrainian) online or by phone interview.

Excluded from the MAP-U study are individuals who cannot be reached on multiple attempts and those who are in military service and perform combat missions as part of the territorial defence, the armed forces of Ukraine or other military formations.

#### MAP-Z

The target population of MAP-Z is adult Ukrainians who fled from Ukraine and currently live in the canton of Zurich. The source population is Ukrainians registered with refugee status S in the canton of Zurich and the study population consists of a random selection of this group of persons conducted by the Swiss Federal Statistical Office (FSO).

Inclusion criteria for the random, age-stratified (same age categories as for MAP-U) sampling are adults (aged ≥18 years) from Ukraine with refugee status S residing in the Canton of Zurich, providing informed consent and able to complete the surveys (available in Ukrainian) online. Exclusion criteria are individuals who cannot be reached on multiple attempts.

For the control group, adults from the general population of the Canton of Zurich will be randomly selected from the resident registry by the Swiss FSO, again age-stratified (same age categories as for MAP-U).

Inclusion criteria for the random sampling are adults (aged ≥18 years) residing in the Canton of Zurich with either Swiss citizenship or residence permit B or C, providing informed consent and able to complete the surveys (available in German) online.

### Study Recruitment and Informed Consent

For MAP-U, potential participants in Ukraine will be informed about the study and recruited by email sent through the electronic database REDCap. Depending on the participation rate, non-responders will be contacted by means of Telegram and phone calls. All potential participants will be provided in REDCap with a video in Ukrainian which includes all relevant information regarding the purpose of the study, study procedures, and institutions involved, and the information of a professional hotline number were persons can find psychological support in case they are emotionally stressed by completing the questionnaire.

Recruitment of participants for both populations in MAP-Z (Ukrainian refugees and general population of Zurich) will be performed similarly. The randomly selected individuals will be informed about the study and recruited via postal mail. The letter will contain information about the study, an invitation to participate and a personalised access code to access REDCap. In REDCap, they will also be provided with a similar video as MAP-U (both in Ukrainian for refugees and German for the general population). Potential participants in both studies will also be able to access a PDF version of the detailed study participant information form.

Those interested will provide their informed consent to participate in the study electronically via REDCap and will be directly forwarded to fill the baseline assessment questionnaires.

### Baseline Assessment

At baseline, primary and secondary outcomes are assessed. Additional information collected through the baseline questionnaire includes oblast, contact information, and sociodemographic information including age, sex, citizenship, marital status, total number of people living in household, household income, residential situation, proximity to combat zone (i.e., previously occupied vs. never occupied, far from or close to combat zone) and details on migration processes and war-related experiences. Additionally, data on pre-existing health conditions including the presence of physical and mental health conditions and health behaviours (e.g., smoking, physical activity, and access to healthcare) will be collected.

### Follow-Up Assessments

Follow-up assessments will take place every 3 months for at least 2 years and include all primary endpoints and selected secondary endpoints.

### Statistical Analysis Plan and Sample Size Calculation

#### Statistical Methods

We will use medians and interquartile ranges or absolute numbers and percentages for the descriptive analyses. For prevalence estimates, there is the risk of overestimation if based on raw numbers since the cut-offs of instruments to classify into mental health condition (yes, likely) are optimized for sensitivity and screening for mental health conditions and thus include varying extent of false-positives. A recent systematic review indicated that prevalence estimates based solely on standardized instruments are about double as high as prevalence estimates based on standardized diagnostic interviews [18]. We will take this challenge into account in different ways: First, we will provide prevalence estimates and 95% credible intervals using a Bayesian logistic regression model adjusted for age-group and cluster of oblasts taking sensitivity and specificity of the instruments, as described above, and their uncertainty into account [33, 34].

We will conduct these analyses for the entire study population and also estimate prevalence of the four mental health conditions for subgroups as defined by cluster of oblasts (see above), age group and sex. The same applies to the analyses of aims 2 (impact of mental health on general health and healthcare seeking behaviour) and 3 (needs, preferences, barriers, and facilitators for mental healthcare services) where it is critical to gain an understanding of differences across subgroups. To assess factors (cluster, age, sex, sociodemographic variables, war-related experiences) associated with alcohol use disorder, abuse, anxiety, depression, or PTSD as well as with general health and healthcare seeking behaviour and needs, preferences, barriers, and facilitators for mental healthcare services we will use regression models while carefully adjusting for potential confounders. We will also consider clustering approaches to identify subgroups of the population with specific mental health burden and healthcare and social support needs. We will conduct all analyses in R, using the most current version at time of analysis https://www.r-project.org/.

#### Sample Size Considerations and Calculation

We expect a prevalence of 15% for alcohol use disorder, 25% for anxiety and depression and 40% for PTSD in the Ukrainian population based on the available prevalence data in Ukraine and on data from other conflict areas [11]. Sample size requirements are highest for a prevalence of 40%, on which we thus base sample size calculations. We consider a specific age group in a specific cluster (e.g., participants 25–44-year-old in a specific cluster of oblasts) as the unit for which we need a certain precision to estimate prevalence. To provide a precision of ±5% in estimating prevalence with 95% confidence intervals for a specific age group in a specific cluster, the required sample size is 369 survey respondents per combination of age group and cluster. Assuming a potential attrition of 15% among those who consent to participate, the required sample size for enrolment is 435 per age and cluster stratum. The size of the target sample is 5,220 persons (net sample; given 4 age and 3 cluster of oblasts strata). Considering a participation rate of approximately 7% for MAP-U, reflecting the difficult situation in Ukraine, the final number to be randomly drawn from the population registry in Ukraine is 1,740 per stratum and 74,570 for the total sample, respectively. For the MAP-Z study, a participation rate of 25% is assumed based on experiences with previous population-based studies in the canton of Zurich (e.g., Corona Immunitas studies). For the age groups of 25–44 and 45–64 years in people living with protection status S as well as for all four age groups of the general population of the canton of Zurich we will also have 435 participants per age group, or 1,740 people to be invited initially. But there are less than 1,740 people with protection status S in the youngest (18–24 years) and oldest (65+ years) age group with protection status S in Zurich. Thus, we will aim for randomly selecting 684 people in those two age groups in order to have 171 (25%) finally included. In these age groups, the precision will be lower, and 95% CI expected at ±8%. The total sample sizes for people living with protection status S and for the general population living in the canton of Zurich will thus be 1220 and 1740, respectively.

This sample size provides 95% confidence intervals of ±6%–7% when analysing the results separately for women and men, age groups and cluster of oblasts. Sample size calculations were performed using the Scalex SP calculator [34].

## Results

Detailed results and analyses of the mental health of the Ukrainian population will be presented and discussed in subsequent publications.

## Discussion

The current war in Ukraine undoubtedly causes enormous suffering, with an estimated 10 million people in Ukraine possibly experiencing adverse mental health effects [8]. The war has triggered an international response and prompted numerous national and international relief and crisis mitigation efforts for mental health. In the absence of a robust mental health monitoring system, it remains uncertain whether such relief efforts effectively support those in need, both now and in the future. Thus, establishing a comprehensive mental health surveillance system is essential to provide actionable insights for policymakers, national and international humanitarian agencies, healthcare providers, and communities.

While starting from solid and practice-tested methodologies and platforms, this study will also create key insights for authorities and scientific communities. This will provide vital learnings about establishing, scaling up and maintaining population-based monitoring studies under adverse conditions - a need that is clear from the lack of pertinent scientific literature and best practices. Furthermore, the monitoring system will enable epidemiologists and mental healthcare providers to leverage technological advances, examine their usefulness and implement novel digital tools to further expand into hard-to-reach subgroups. From the perspective of implementation science for digital health, the MAP study will provide novel insights into the acceptability, usability, scalability, and efficiency of conducting large scale, representative digital mental health surveillance effort during an ongoing war-time situation. Our study protocols and tools will directly inform needed best-practices for similar endeavours and provide guidance for epidemiologists and health researchers around the world seeking to repeatedly measure mental health outcomes in crisis situations (e.g., political conflict, mass migrations, climate disasters) by use of open-source tools.

Through our strong, existing collaboration between Swiss, Ukrainian and international scientists and policymakers, the necessary science-to-policy communication lines are in place to use our results – as they become available, uploaded to the online platform – to inform and guide decision-makers worldwide (i.e., public health officials, NGOs, humanitarian organizations).

Data on the prevalence of the four mental health conditions can serve as a basis for evidence-based policymaking and service implementation. Local, regional, and national policymakers will be better equipped to make informed decisions regarding resource allocation to regions in need of increased mental health support or to population groups requiring tailored mental health services. International humanitarian organizations operating in Ukraine can use our results to appropriately channel mental health services to target populations and (re)allocate funding for mental health spending. MAP findings can contribute to improving access to mental health services, as results can inform planning new mental health programs and expansion or adaptation of existing programs according to population needs. Results can also support the development of specialized training programs for professionals, especially in war and post-war areas. Furthermore, MAP will foster community support and promote mental health awareness. Reducing stigma around mental health is crucial to improve support and access to health services during and after the war.
